# Evaluation of in vitro anti-inflammatory effects of crude ginger and rosemary extracts obtained through supercritical CO_2_ extraction on macrophage and tumor cell line: the influence of vehicle type

**DOI:** 10.1186/s12906-015-0896-9

**Published:** 2015-10-29

**Authors:** Oselys Rodriguez Justo, Patricia Ucelli Simioni, Dirce Lima Gabriel, Wirla Maria da Silva Cunha Tamashiro, Paulo de Tarso Vieira Rosa, Ângela Maria Moraes

**Affiliations:** Department of Engineering of Materials and of Bioprocesses - School of Chemical Engineering, University of Campinas, 13083-852 Campinas, SP Brazil; Department of Genetics, Evolution and Bioagents - Institute of Biology, University of Campinas, 13083-970 Campinas, SP Brazil; Departament of Physical Chemistry - Institute of Chemistry, University of Campinas, 13083-970 Campinas, SP Brazil

**Keywords:** Ginger, Rosemary, Anti-inflammatory, Macrophages, J774 cell, Nitric oxide, Antioxidant, Liposome, DMSO, Pluronic F-68

## Abstract

**Background:**

Numerous plants from have been investigated due to their anti-inflammatory activity and, among then, extracts or components of ginger (*Zingiber officinale* Roscoe) and rosemary (*Rosmarinus officinalis* L.), sources of polyphenolic compounds. 6-gingerol from ginger rhizome and carnosic acid and carnosol from rosemary leaves present anti-tumor, anti-inflammatory and antioxidant activities. However, the evaluation of the mechanisms of action of these and other plant extracts is limited due to their high hydrophobicity. Dimethylsulfoxide (DMSO) is commonly used as a vehicle of liposoluble materials to mammalian cells in vitro, presenting enhanced cell penetration. Liposomes are also able to efficiently deliver agents to mammalian cells, being capable to incorporate in their structure not only hydrophobic molecules, but also hydrophilic and amphiphilic compounds. Another strategy is based on the use of Pluronic F-68, a biocompatible low-foaming, non-ionic surfactant, to disperse hydrophobic components. Here, these three delivery approaches were compared to analyze their influence on the in vitro anti-inflammatory effects of ginger and rosemary extracts, at different concentrations, on primary mammalian cells and on a tumor cell line.

**Methods:**

Ginger and rosemary extracts free of organic solvents were obtained by supercritical fluid extraction and dispersed in DMSO, Pluronic F-68 or liposomes, in variable concentrations. Cell viability, production of inflammatory mediators and nitric oxide (NO) release were measured in vitro on J774 cell line and murine macrophages primary culture stimulated with bacterial lipopolysaccharide and interferon-γ after being exposed or not to these extracts.

**Results:**

Ginger and rosemary extracts obtained by supercritical CO_2_ extraction inhibited the production of pro-inflammatory cytokines and the release of NO by peritoneal macrophages and J774 cells. The delivery vehicles influenced the anti-inflammatory effects. Comparatively, the ginger extract showed the highest anti-inflammatory activity on the tumor cell line. Controversially, rosemary extract dispersed on DMSO induced a more significant IL-1 and TNF-α reduction than ginger extract in primary macrophages.

**Conclusions:**

Amongst the tested delivery vehicles, DMSO was the most suitable, presenting reduced cytotoxicity, followed by Pluronic F-68 and liposomes, provably due to differences in their form of absorption, distribution and cellular metabolism. Co-administration of liposomes and plant extracts may cause death of macrophages cells and induction of NO production. It can be concluded that some of the beneficial effects attributed to extracts of ginger and rosemary may be associated with the inhibition of inflammatory mediators due to their high antioxidant activity. However, these effects were influenced by the type of delivery vehicle.

## Background

Several diseases, such as cancer, vascular diseases (atherosclerosis, stroke, heart failure, and cerebrovascular disease), metabolic diseases (diabetes and metabolic syndrome), neurological diseases (Parkinson’s disease, Alzheimer’s disease, epilepsy and dementia), pulmonary diseases, rheumatoid arthritis, osteoarthritis, muscular dystrophy and chronic fatigue syndrome are associated with inflammation processes. Inflammation is a complex host response to injury, involving the recruitment of leukocytes and extravasation of plasma proteins. This process is coordinated by a range of chemical mediators, such as arachidonic acid metabolites, cytokines and nitric oxide (NO) [1–4].

Traditionally in clinical practice, treatment of inflammatory disorders includes the extensive use of non-steroidal anti-inflammatory drugs (NSAIDs) and corticosteroids. The action of these therapeutic agents ranges from inhibition of enzymes responsible for production of arachidonic acid metabolites to inhibition of cytokines expression. However, these medicines have some adverse effects that limit their use, such as gastrointestinal damage, mainly when administered at high doses for prolonged periods [5].

In recent years, numerous products derived from plants have been investigated for therapeutic application due to their pharmacological activity on inflammatory processes and other illnesses. Dietary agents can interfere with several cell-signaling pathways and the molecular targets modulated may be the basis for how these compounds not only prevent but also treat the diseases [5]. Many plants, herbs and spices typically used for food flavoring and nutrition are excellent sources of phenolic compounds, which have been reported to show good antioxidant activity. Frequently the anti-inflammatory activity of natural compounds has been associated to their antioxidant activity, but their capacity to suppress nitric oxide production is also emphasized [6–8].

Ginger (*Zingiber officinale* Roscoe) rhizomes and rosemary (*Rosmarinus officinalis* L.) leaves are among the most important and extensively used spices worldwide. Extracts or components from ginger and rosemary, such as polyphenolic compounds (6-gingerol and its derivatives for ginger rhizome, as well as carnosic acid and carnosol for rosemary leaves) have received special attention, specifically for their anti-inflammatory, antitumor and antioxidant activities [7, 9–14].

Animal cell culture studies are useful for elucidating the mechanisms of action of plant extracts. But, these evaluations are often limited due to the high hydrophobicity of the plant extracts, their sensitivity to heat, light, oxygen, and their inherent poor bioavailability. In these studies, organic compounds such as ethanol, methanol, ethyl acetate, tetrahydrofuran, dimethylsulfoxide (DMSO), dichloromethane and carboxy methylcellulose are commonly used as vehicles to deliver the liposoluble materials to the cells. DMSO stands out in this type of application, because this polar and aprotic solvent is able to dissolve an enormous range of polar and nonpolar small molecules, being, in addition, miscible with water. Its uses encompass cells, tissue and organ preservation as well as enhancement of pharmaceutical agent penetration.

While plant extracts or their components are recommended for prevention of inflammatory process and other diseases, the use of solvents or detergents to disperse hydrophobic active molecules during cell culture or animal testing has been questioned [15, 16]. In this sense, the design of adequate systems to protect, carry, deliver and control the release of lipophilic bioactive molecules extracted from plants is of paramount importance to proper analyze their pharmacological effects.

Nanoparticles, such as liposomes or lipid vesicles, have proved themselves as excellent systems for medical applications ranging from diagnostics to controlled drug delivery. Liposomes are able to efficiently incorporate hydrophobic, hydrophilic and amphiphilic molecules, being then useful as vehicles for the administration of species with different characteristics. These vesicles may be obtained reproducibly and with costs relatively low through the use of methodologies which do not require harmful organic solvents, and as a consequence, inherent vehicle toxicity may then be significantly reduced when assessing in vitro and in vivo effects of plant extracts [17, 18].

Another strategy useful to disperse hydrophobic components is their solubilization with the Pluronic F-68 (PF-68), also known as Lutrol VR F68 or Poloxamer 188, a triblock copolymer composed of poly(ethylene oxide)-poly(propylene oxide)-poly(ethylene oxide) (PEO-PPO-PEO). PF-68 is a low-foaming, non-ionic surfactant that has multiple functional effects on animal cells [19, 20], being widely used to protect the cells from shear stress during culture in stirred tanks.

Thus, the purpose of the present study was to evaluate and compare the in vitro effects of ginger and rosemary extracts obtained by supercritical fluid extraction, a technology that renders preparations free of toxic organic solvents, using liposomes, Pluronic-F-68 and DMSO as vehicles. The production of inflammatory mediators, specifically cytokines and nitric oxide, by two different types of macrophage cells was analyzed. To our knowledge, no other study has been carried out to compare the performance of DMSO, Pluronic-F-68 and liposomes as delivery systems for the in vitro evaluation of these plant extracts.

## Methods

### Cells

Two types of cells were used, freshly extracted mouse peritoneal macrophages and cells of the tumor J774 lineage. Cell line J774 (ATCC) was donated by Dr. Michael Palladino (Genentech), being a macrophage-like mouse cell line originally isolated from reticulum cell sarcoma [21]. Interleukin 1β is synthesized continuously by this cell line, which is then commonly used as a tool to test anti-inflammatory agents [22, 23]. Murine cells were collected from 8 weeks old male or female BALB/c mice obtained from the Multi-Institutional Center for Biological Investigation (CEMIB) of the University of Campinas (UNICAMP), Brazil. The mice were maintained in a temperature-controlled room under pathogen-free conditions and received a diet of autoclaved food and water. The experimental protocol was approved by the institutional Committee for Ethics on the Use of Animals (CEUA/UNICAMP, 1854-1). The thyoglycolate-elicited murine macrophages were harvested from the peritoneal cavities of BALB/c mice through intraperitoneal injection of 3 mL of 3 % sodium thioglycollate medium 4 days before the assays. The cells were transferred to sterile polypropylene conical centrifuge tubes kept on ice and centrifuged at 200 g/min for 10 min at 5 °C. The supernatant was discarded and the sediment was ressuspended in DMEM supplemented with 10 % fetal bovine serum (FBS; complete medium). Cells were counted in hemocytometer and the suspension was adjusted to 1 × 10^6^ cells/mL. J774 cells preserved in liquid nitrogen were reactivated and cultured until semi-confluence in complete DMEM medium in a humidified incubator at a 5 % CO_2_, at 37 °C. Then, these cells were harvested, washed with DMEM without FBS, and their concentration was adjusted to 1 × 10^6^ cells/mL with complete medium.

### Reagents

Lipopolysaccharide (LPS, *Escherichia coli*, O111:B4), 3-(4,5-dimethylthiazol-2-yl)-2,5-diphenyltetrazolium bromide (MTT), Dulbecco's Modified Eagle medium (DMEM), dipalmitoylphosphatidylcholine (DPPC), cholesterol (Chol), dimethylsulfoxide (DMSO) and Pluronic F-68 were obtained from Sigma-Aldrich Co. (St. Louis, MO, USA). Fetal bovine serum was purchased from Cultilab (Campinas, Brazil). All reagents used were of at least analytical grade.

### Plant extracts

Ginger (*Zingiber officinale* Roscoe) and rosemary (*Rosmarinus officinalis* L.) were attained from two Brazilian organic herbarium sources: the Experimental Farm of Lageado at the State University of São Paulo (UNESP), Botucatu - São Paulo and the Agronomic Institute of Campinas (IAC), Campinas - São Paulo, respectively. The raw materials (ginger rhizome and rosemary leaves) were dried at room temperature during 3 days, triturated in a rotor mill (TE090, Tecnal, Brazil), packed in plastic bags and kept at -5 °C. The extracts of the plants were obtained by supercritical fluid extraction (SFE) and characterized as described in detail by Justo et al. [24]. An Applied Separations unit (Spe-ed SFE, model 7071, Allentown, USA) was used, with an extractor vessel (Thar Designs, Pittsburgh, USA) of total volume equal to 300x10^−6^ m^3^ (0.1286 m in height, with an internal diameter of 0.0545 m). The ginger and rosemary extractions were carried out using 1.42x10^−4^ kg/s of CO_2_, at 30 °C and 30 MPa and 1.13x10^−4^ kg/s of CO_2_ at 40 °C and 25 MPa, respectively, reaching global yields of 2.4 and 2.9 % for ginger and rosemary, correspondingly. Analysis of the extracts by high-performance liquid chromatography (HPLC), gas chromatography coupled to mass spectrometry (GC-MS) and regarding both phenolic compounds content and antioxidant activity were described by Justo et al. [24]. Before their use in cell culture experiments, the extracts were dispersed in DMSO (≥99.9 %), Pluronic F-68 (0.1 %) or in lipid vesicles (liposomes). Pluronic® F-68 was used at a working concentration of 0.1 % after dilution with cell culture grade water. All preparations were sterilized by filtration through 0.2 μm pore-size filters and stored at -20 °C.

### Preparation of liposomes

Liposomes were prepared by the ethanol injection as described elsewhere [25]. The vesicles were obtained by the injection of the lipids (100 mmol/L of DPPC:Chol, 60:40 mol %) mixed with the plant extracts solubilized in ethanol at 25 °C through a syringe pump (model ST-670 T, Samtronic) coupled to a 4 μm (inside diameter) stainless-steel needle into HEPES buffer (10 mmol/L and pH 7.4). A ratio of 4 volumes of organic solution to 76 volumes of aqueous solution was used. While injecting the alcoholic solution and 3 min afterwards, the mixture, kept at 25 °C, was sonicated in a bench sonicator.

The phospholipid concentration of liposomal samples was determined using modifications of a total phosphate spectrophotometric assay technique [26], employing a Beckman DU 640 UV-visible spectrophotometer. The molar concentration of total lipid was calculated dividing the phospholipid concentration determined using the phosphate assay by the mole fraction of phospholipid in the vesicle preparation (equal to 0.6), to account for the presence of cholesterol.

Absorbance measurements at 280 nm were used to calculate the concentration of the extracts dispersed in the liposomes. Lipid vesicles (20 μL) were disrupted with methanol (0.5 mL), agitated, and after 1 h, absorbance was read at 280 nm, a wavelength typically used to analyze phenolic compounds commonly present in both plant extracts. The calibration curves obtained after deducting the contribution of the liposomes were linear from 0 to 0.17 g/L for ginger extract and from 0 to 0.54 g/L for rosemary extract. Each experiment was performed at least in triplicate.

### Cell culture

The murine peritoneal macrophages and the J774 cells suspension with concentrations adjusted to 1 × 10^6^ cells/mL were seeded at a density of 2 × 10^5^ cells/well in 96-well plates (100 μL per well) and cultured for 24 h. Dead cells and debris were then removed by washing with DMEM without FBS. Following, 50 μL/well of either DMEM medium containing 10 % heat inactivated fetal calf serum (FCS; Cultilab, Brazil) or the plant extracts (in the different tested dispersants) diluted in culture medium were added to the remaining adherent cells, and the plates were incubated for 1 h in the same culture conditions. Different concentrations of ginger and rosemary extracts were tested to verify their influence on the in vitro anti-inflammatory effects on primary mammalian cells and on tumor cell line. Subsequently, the wells were filled with 50 μL of culture medium or a solution of LPS/IFN-γ (1 μg/mL/150 IU/mL). The plates were incubated for additional 48 h before being used for the proliferation, NO and cytokines assays. Cell proliferation was examined using a colorimetric assay based on the MTT reagent. An aliquot of 10 μL of MTT (5 mg/mL) dissolved in 0.02 M phosphate buffered saline (PBS) at pH 7.0 was added to each well containing cells in culture and the plates were then incubated for 4 h at 37 °C. The formazan crystals formed by MTT reduction by living cells were dissolved in 5 % SDS in 0.01 N HCl solution and the optical density was measured using a Multiskan MS microplate reader (Labsystems Oy, Helsink, Finland) at 540 nm. Each experiment was performed at least in triplicate.

### Measurement of nitrite concentration

Nitric oxide (NO) released into the supernatants of the cell cultures was indirectly determined using a quantitative colorimetric assay based on Griess reaction. Aliquots of 50 μL of cell culture supernatants were incubated with 50 μL of Griess reagent, consisting of 1 % sulfanilamide, 0.1 % N-(1-naphthylethylenediamine) dihydrochloride and 2.5 % ortho-phosphoric acid. After 10 min at room temperature, the optical density was measured in a Multiskan MS microplate reader (Labsystems Oy, Helsink, Finland) at 540 nm. Nitrite concentrations were calculated by comparison to a calibration curve obtained with sodium nitrite standard solutions. Each experiment was performed at least in triplicate.

### Cytokine determination

Enzyme-linked immunosorbent assay (ELISA) kits (BD-OptEIA Mouse Set; BD Biosciences Pharmingen, San Diego, CA) were used for interleukin (IL)-1 and tumor necrosis factor (TNF)-α cytokine determination in the culture supernatants. The kits were used following the recommendations of the manufacturer.

### Statistical analysis

Statistical comparisons were performed by one-way ANOVA followed by post hoc Bonferroni’s test. The software Prism (version 5.0, Graph Software, Inc. San Diego, CA, USA) was used for the statistical analysis. The data are presented as the mean ± standard error of the mean (S.E.M.). Probability (*p*) values lower than 0.05 were considered statistically significant.

## Results

### Antioxidant activity and content of phenolic compounds of the extracts

The ginger and rosemary extracts obtained by supercritical CO_2_ extraction exhibited high contents of phenolic compounds (136.1 and 62.7 mg gallic acid equivalent per gram of sample, for ginger and rosemary, respectively). The main active compounds identified were 6-gingerol and carnosic acid, for ginger and rosemary, correspondingly. Also, high free radical scavenging capacity was observed for both extracts against the free radicals 2,2′-azinobis (3-ethylbenzothiazoline-6-sulfonate) (ABTS^•+^) (350 and 200 mM Trolox equivalent/g extract, for ginger and rosemary, respectively) and 2,2′-diphenyl-1-picrylhydrazyl (DPPH^•^) (145 and 80 mM Trolox/g extract, for ginger and rosemary, respectively), as described by Justo et al. [24].

### Evaluation of cytotoxicity and NO production of different extracts or solvent/delivery systems on murine macrophages cells

In order to determine if extracts and solvent/delivery systems have cytotoxicity or inflammatory effects, different concentrations of extracts were tested on non-stimulated macrophages or J774 cells. These assays allowed to determine the adequate concentration of extract and solvent system and to exclude the possibility that the observed effects of the extracts were due to cytotoxicity.

**Fig. 1:**
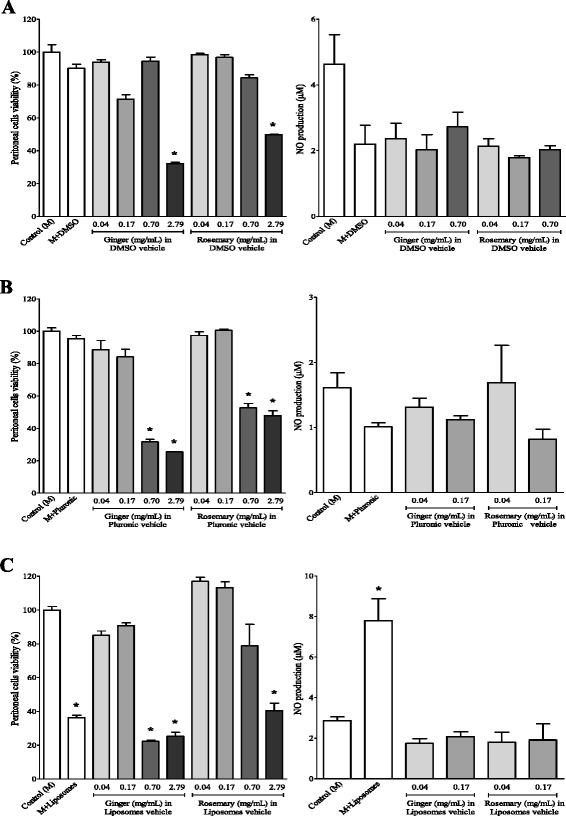
Effects of ginger and rosemary extracts on the viability and nitric oxide production by non-stimulated murine peritoneal macrophages cells: influence of vehicle and concentration of extracts. Cells were incubated for 48 h with different concentration of ginger and rosemary extracts dissolved in DMSO (**a**), Pluronic-F68 (**b**) and liposomes (**c**). Cells were treated with extracts at the indicated concentrations. Cell viability was determined by MTT assay. The amount of NO released into the culture supernatants is expressed as nitrite. The columns represent the means ± SEM of the data from triplicate tests. * indicates data statistically significantly different in comparison with the control (no-treated cells) at *p* < 0.05

As shown in Fig. 1, DMSO did not affect significantly the proliferation of murine peritoneal macrophages cells. Only at 2.79 mg/mL ginger and rosemary extracts dissolved in DMSO showed high cytotoxic effects to peritoneal macrophages without inflammatory stimuli (Fig. 1a). Since both ginger and rosemary extracts dispersed in DMSO showed cytotoxicity only at high doses, the NO production investigation was limited to extract concentrations lower than 2.79 mg/mL, in which significant cell death was not observed. The analysis of NO production revealed that neither ginger nor rosemary extracts dissolved in DMSO were able to induce NO production in peritoneal macrophages without inflammatory stimuli (Fig. 1a) above that of the control.

When dissolved in Pluronic F-68, none of the extracts resulted in inhibition of proliferation at concentrations of 0.04 and 0.17 mg/mL, whereas significant cell death was noted when these extracts were used at 0.7 and 2.79 mg/mL. As the extracts dispersed in Pluronic F-68 were cytotoxic at high doses, the NO production was analyzed only at concentrations lower than 0.7 mg/mL. Also in this study, the exposure to ginger and rosemary extracts in Pluronic F-68 did not induce NO production by these cells in levels significantly higher that of the control (Fig. 1b).

When murine peritoneal macrophages were cultured in the presence of ginger and rosemary extracts dispersed in liposomes, the nanoparticles showed to be cytotoxic if not associated to the apparently protective effect of the extracts (Fig. 1c). However, a significant decrease on the proliferation of peritoneal macrophages was observed for formulations consisting of ginger and rosemary extracts dispersed in liposomes when the highest concentration (2.79 mg/mL) was used. In this condition, when compared to the control, cell proliferation was reduced to around 75 % (ginger extract) and 60 % (rosemary extract). In contrast, exposure of these peritoneal cells to rosemary extract at 0.04 and 0.17 mg/mL for 48 h did not result in significant adverse effects on cell proliferation. At the same concentrations, the ginger extract had moderate effect on cell proliferation, which reached values of 15 and 9 % higher than the control.

Since the ginger and rosemary extracts dispersed in liposomes showed cytotoxicity at high doses, the NO production was analyzed only at concentrations lower than 0.7 mg/mL. In these conditions, measurable NO production levels were not detected. However, liposomes were capable of inducing the production of this molecule at relatively high levels in comparison to the control.

**Fig. 2:**
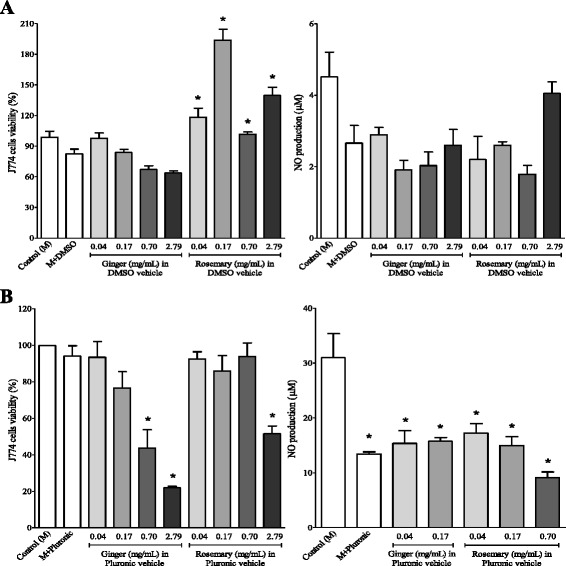
Effects of ginger and rosemary extracts on the viability and nitric oxide production by non-stimulated J774 macrophages cells: influence of vehicle and concentration of extracts. Cells were incubated for 48 h with different concentration of ginger and rosemary extracts dissolved in DMSO (**a**) and Pluronic-F68 (**b**). Cells were treated with extracts at the indicated concentrations. Cell viability was determined by MTT assay. The amount of NO released into the culture supernatants is expressed as nitrite. The columns represent the means ± SEM of the data from triplicate tests. * indicates data statistically significantly different in comparison with the control (no-treated cells) at *p* < 0.05

As shown in Fig. 2a, DMSO did not significantly affect the proliferation of murine J774 cell line. No statically significant toxic effects were observed when exposing J774 cells to ginger or rosemary extracts dissolved in DMSO in comparison to the results attained for control cells. In fact, cell growth seemed to be stimulated by the rosemary extract at all tested concentrations. Nitric oxide production level above the control experiment was not observed in the tested conditions.

Different amounts of ginger and rosemary extracts dissolved in Pluronic F-68 were also added to murine J774 cell cultures. As depicted in Fig. 2b, Pluronic-F-68 itself did not show any cytotoxic effect to this cell line. However, when J774 cells were exposed to the two highest concentrations of ginger extract (0.7 and 2.79 mg/mL) or to the highest concentration of rosemary (2.79 mg/mL) extract, a significant reduction on cell proliferation was observed. The exposure to ginger and rosemary extracts dissolved in Pluronic F-68 at concentrations lower than 2.79 mg/mL resulted, in the absence of inflammatory stimuli, in the production of low amounts of NO in comparison to the control experiment, and the same was noticed when only Pluronic-F68 was added. As a general trend, J774 cells seemed less sensitive to the effects of DMSO and Pluronic F-68 vehicles than murine peritoneal macrophages. Given that liposomes proved to be more cytotoxic than DMSO and Pluronic F-68, this vehicle was not used in this part of the study.

### Evaluation of the inhibitory effects of ginger and rosemary extracts on LPS + INF-γ stimulated macrophage cells

The anti-inflammatory effects of ginger or rosemary extracts dispersed in the different solvent/delivery approach were evaluated in vitro in primary and adherent cell line stimulated with LPS + INF-γ stimuli. Also, we tested several doses of the extracts in three different solvents/ delivery systems in order to verify the best concentration of each system.

**Fig. 3:**
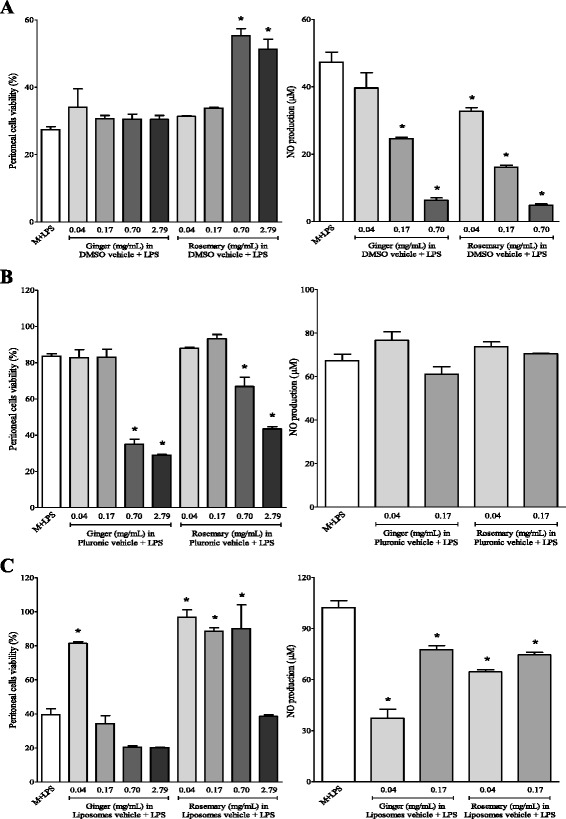
Effects of ginger and rosemary extracts on the viability and nitric oxide production by LPS and/or IFN-γ-stimulated murine peritoneal macrophages cells: influence of vehicle and concentration of extracts. Cells were incubated for 48 h with ginger and rosemary extracts dissolved in DMSO (**a**), Pluronic-F68 (**b**) and liposomes (**c**) and stimulated with LPS/IFN-γ: LPS (1 μg/mL) plus IFN-γ (150 IU/mL). Cells were treated with extracts at the indicated concentrations. Cell viability was determined by MTT assay. The amount of NO released into the culture supernatants is expressed as nitrite. The columns represent the means ± SEM of the data from triplicate tests. * indicates data statistically significantly different in comparison with the control (no-treated cells) at *p* < 0.05

Nitric oxide production was measured in peritoneal macrophages and J774 cells treated with extracts in tested vehicles, as described below. In the presence of LPS + INF-γ, rosemary extract at 0.7 and 2.79 mg/mL in DMSO was able to circumvent partially cell death induced by the inflammatory stimuli in peritoneal macrophages (Fig. 3a). Also, ginger and rosemary extracts dissolved in DMSO at concentrations from 0.04 to 0.7 mg/mL inhibited the production of nitric oxide induced by LPS plus IFN-γ in a dose-dependent manner. The levels of NO increased in the presence of LPS plus IFN-γ stimulation to 47.3 μmol in comparison to control cells (4.6 μmol).

Extracts dissolved in Pluronic F-68 resulted in significant proliferation inhibition at concentrations of 0.7 and 2.79 mg/mL in the cells activated with LPS, whereas none of the extracts caused cell death when used at 0.04 and 0.17 mg/mL (Fig. 3b). Peritoneal macrophages produced large amounts of NO when activated with LPS plus IFN-γ. However, neither ginger nor rosemary extracts dissolved in Pluronic F-68 were able to inhibit NO production induced by LPS/IFN-γ. The levels of NO increased in the presence of LPS plus IFN-γ stimulation to 67.4 μmol in comparison to control cells (1.6 μmol).

When peritoneal cells were stimulated with lipopolysaccharide (LPS) and interferon (IFN-γ), the results revealed that ginger and rosemary extracts dispersed in liposomes, respectively at concentrations equal to 0.04 mg/mL and up to 0.7 mg/mL, exerted protective effects regarding cell death (Fig. 3c). The levels of NO increased in the presence of LPS plus IFN-γ stimulation to 102.3 μmol in comparison to control cells (2.9 μmol). The results showed that ginger and rosemary extracts dispersed in liposomes exhibited statistically significant inhibitory effects on NO production induced by LPS plus IFN-γ. As the ginger and rosemary extracts dispersed in lipid vesicles showed cytotoxicity only at concentration higher that 0.17 mg/mL, the inhibition of NO production at concentrations lower than 0.7 mg/mL was not due to cell death. In these cases, the cytotoxic effects might be associated with the vehicle used to disperse the extracts, since it was also diluted in the assays in the same proportion as the extracts.

**Fig. 4:**
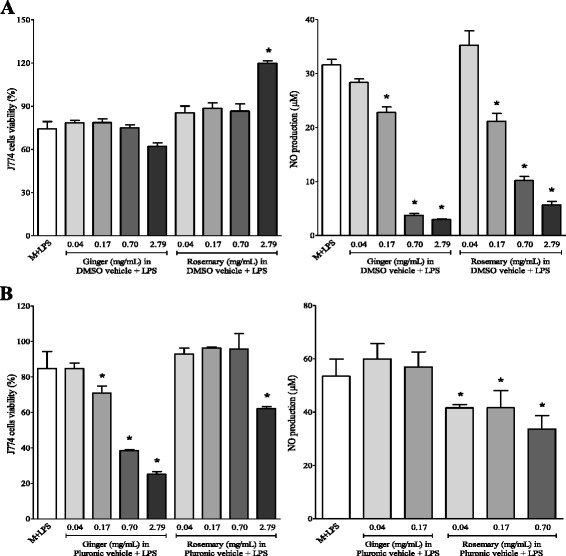
Effects of ginger and rosemary extracts on the viability and nitric oxide production by LPS and/or IFN-γ-stimulated J774 macrophages cells: influence of vehicle and concentration of extracts. Cells were incubated for 48 h with ginger and rosemary extracts dissolved in DMSO (**a**) and Pluronic-F68 (**b**) and stimulated with LPS/IFN-γ: LPS (1 μg/mL) plus IFN-γ (150 IU/mL). Cells were treated with extracts at the indicated concentrations. Cell viability was determined by MTT assay. The amount of NO released into the culture supernatants is expressed as nitrite. The columns represent the means ± SEM of the data from triplicate tests. * indicates data statistically significantly different in comparison with the control (no-treated cells) at *p* < 0.05

As depicted in Fig. 4a, treatment of J774 cells with LPS + IFN-γ did not result in significant proliferation decline when DMSO was used as a vehicle. Treatment of J774 cells with ginger and rosemary extracts dissolved in DMSO inhibited LPS-induced NO production in a dose-dependent manner at concentration range from 0.04 to 2.79 mg/mL. Treatment of J774 cells with LPS + IFN-γ and ginger extract dissolved in Pluronic-F68 caused a significant proliferation decline in a dose-dependent manner at concentration range from 0.17 to 2.79 mg/mL, whereas significant cell death was noted only when J774 cells were exposed to the highest concentration of rosemary (2.79 mg/mL) extract (Fig. 4b). The exposure of J774 cells to LPS plus IFN-γ resulted in the production of high amounts of NO, which was inhibited only by rosemary extract dispersed in Pluronic F-68.

### Comparison of anti-inflammatory effects of ginger and rosemary extracts dissolved in DMSO: inhibition of pro-inflammatory cytokines

**Fig. 5:**
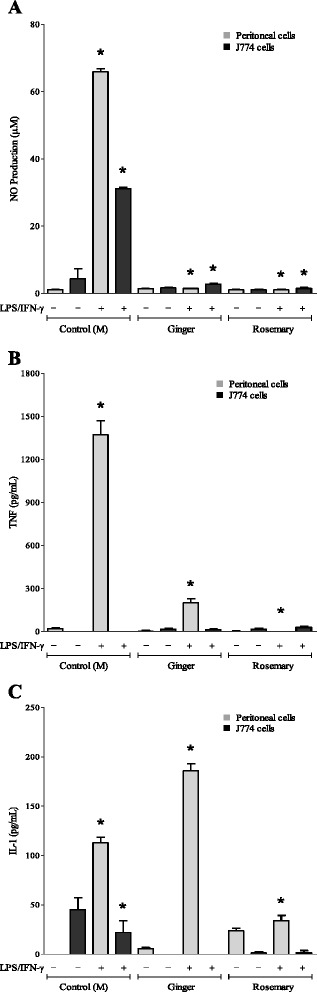
Comparison of the effects of ginger and rosemary extracts dissolved in DMSO on nitric oxide release and inflammatory cytokine production by stimulated murine peritoneal and J774 macrophages cells. Nitric oxide production (**a**) and levels of TNF-α (**b**) and IL-1 (**c**) cytokines secreted by murine peritoneal or J774 macrophages cells incubated for 48 h with rosemary and ginger extracts dissolved in DMSO and stimulated or not with LPS/IFN-γ: LPS (1 μg/mL) plus IFN- γ (150 IU/mL). Cells were treated with extracts at 0.7 mg/mL. The amount of NO released into the culture supernatants is expressed as nitrite. The columns represent the means ± SEM (*n* = 6). * indicates data statistically significantly different in comparison with the control (no-treated cells) at *p* < 0.05

Pro-inflammatory cytokines stimulate the generation of reactive oxygen and nitrogen species [27]. Therefore, the production of such reactive species in macrophages is likely to be inhibited when the generation of pro-inflammatory cytokines is reduced. Since the tested ginger and rosemary extracts dispersed in DMSO induced the highest inhibition on NO production in specific conditions, the effect of these extracts in DMSO on pro-inflammatory cytokine production by peritoneal macrophages and J774 cells was evaluated (Fig. 5). For the IL-1 and TNF-α assays, the plant extracts were used only at concentrations in which cell proliferation (evaluated by the MTT assay) was not affected (equal to 0.7 mg/mL) to exclude the possibility that the observed effects of the extracts were due to cytotoxicity.

Macrophage cultures showed significant levels of nitrite (NO) in the supernatants when exposed to LPS plus IFN-γ (65.97 μM to stimulated macrophages and 31.13 μM to J774 cells). When used at 0.7 mg/mL, both ginger and rosemary extracts dissolved in DMSO were capable to inhibit the LPS + IFN-induced NO production (Fig. 5a).

An increase in TNF-α production by around 70-fold was observed in murine macrophages treated with LPS plus IFN-γ (1,372.19 pg/mL) in comparison to control cells (19.69 pg/mL), whereas in J774 cells this cytokine was not detected (Fig. 5b). Peritoneal macrophages presented low TNF-α secretion after exposure to the extracts.

Regarding IL-1 production (Fig. 5c), it was enhanced in peritoneal macrophages cultured in the presence of LPS plus IFN-γ (112.93 pg/mL) in comparison to control cells (undetected levels). On the other hand, J774 cells presented a basal production of IL-1 (45.36 pg/mL) that decreased when these cells were cultured with LPS plus IFN-γ (22.07 pg/mL). Only the rosemary extract was able to significantly reduce the production of IL-1 in peritoneal macrophages cultured in the presence of LPS/IFN-γ.

## Discussion

Aiming at the determination of an adequate vehicle to deliver the ginger and rosemary extracts to macrophage cells in culture, DMSO, Pluronic F-68 and liposomes were evaluated in this study. In the present study, it was observed that DMSO was the most adequate vehicle for the tested plant extracts. DMSO has been recognized to have the ability to be an effective carrier of small molecules through a variety of barriers. This solvent can be employed to dissolve organic compounds that are required in animal cell culture media due to its excellent solvating power. Its efficiency as a solvent in the study of the antioxidant properties of lipophilic compounds has been also demonstrated [28].

Probably, the superior effect of DMSO containing-extracts can be attributed to a higher accumulation of the plant compounds in the cells induced by this solvent than by liposomes, which would require adsorption, fusion, endocytosis or leakage of the liposomal contents to cell vicinities, or with Pluronic F-68, which can absorb to the surface of the cells, somehow impairing the diffusion of the extract components to the cell interior. The transport of a particular compound through a given cell membrane is affected mainly by the concentration of the compound in the vehicle, the diffusion coefficient of the compound through the membrane, the partition coefficient of the compound between the membrane and the vehicle, and the thickness of the membrane barrier. DMSO, specially, can have aprotic interactions with intercellular lipids, being able to cause reversible distortion of lipid head groups, which in turn could produce a more permeable packing arrangement [29].

Moreover, when used as a delivery system, DMSO may alter in vitro ginger and rosemary extracts bioavailability and cytotoxicity. At very small and non-lethal concentrations, DMSO can be an exogenous antioxidant due to its ability to react with free radicals [30], acting as a radical scavenger that protects cells against compounds that increase intracellular levels of radical oxygen species [31]. This behavior, despite being desirable, may affect the analysis of potential pharmacological effectiveness of hydrophobic compounds in vitro.

Given that the delivery of plant extracts to cell cultures is generally difficult due to their predominantly hydrophobic nature, organic solvents, serum lipoproteins and surfactants are frequently used to disperse them. Alternatively, plant extracts may also be delivered incorporated in liposomes or in other types of particulate vehicles. Our results also demonstrate that the co-administration of liposomes and plant extracts may cause death of macrophages cells and induction of NO production. It is unclear why the lipid vesicles used in this work were cytotoxic to macrophage cells, but this fact might be related to their lipid composition or to the fact that liposomes can be rapidly phagocytized by macrophages. The lipid vesicles were prepared using DPPC and cholesterol, which could go through oxidation and hydrolysis during cell culture, causing alterations in physicochemical properties of the dispersion such as pH, zeta potential, and phase behavior [32], which could be detrimental to sensitive cell lines.

Abnormalities in lipid metabolism may cause an overproduction of reactive oxygen species and as a result, damage to cell components, including proteins, carbohydrates, nucleic acids, and lipids, might occur, leading to progressive decline in physiological function and ultimately, to cell death [33]. Also, when in excess, cholesterol can be a potent inducer of death on macrophage cells in cultured [34]. In this sense, Takano et al. [35] reported that liposomes could be cytotoxic to the mouse macrophage-like RAW264.7 cell line. These authors proposed that liposome cytotoxicity could be attributed to generation of reactive oxygen species (ROS), required for the induction of apoptosis, being clearly dependent on the cholesterol concentration in the liposomal formulation.

The search for compounds of natural origin with high anti-inflammatory activity, low toxicity, and low cost is of great interest for the development of new treatments to inflammatory diseases. In the last years, several products derived from plants with therapeutic activity on inflammatory processes have been investigated, and frequently extracts with high antioxidant activity are employed for this purpose. In this context, ginger and rosemary extracts have been traditionally used in the popular medicine for a wide range of health-related problems, including chronic inflammatory diseases.

The majority of phytochemicals with anti-oxidative and anti-inflammatory activities so far identified belong to the vast family of polyphenols. Despite the fact that relatively high concentrations may be required to reach the desired therapeutic effect regarding anti-inflammatory activity, great importance is given to the quality of the extract [36]. Ideally, these extracts should be obtained from organically grown plants to avoid the presence of deleterious contaminants as pesticides and through technologies that protect the many labile components of these extracts from degradation during processing. However, traditional organic solvent extraction results in the presence of residual solvent and low purity of the extract. Avoidance of those issues is possible with the use of extraction procedures based on supercritical carbon dioxide technology.

In the present study, the effects of ginger and rosemary extracts on murine macrophages and on a macrophage tumor cell line were analyzed. In vitro studies involving animal and tumor cells are a fast and convenient tool for development of new drugs. Macrophages can initiate inflammatory response and these cells have been commonly used as models in studies for elucidating the anti-inflammatory mechanisms of many plants and herbal extracts [37]. Specifically, the mouse macrophage tumor J774 cell line, first described by Ralph and Nakoinz [38], consists of adherent slow-migrating monocyte-macrophages with the ability to phagocyte or kill foreign cells. This cell line has been widely used as a model for studying new potential drugs on various aspects of macrophage functions, being an effective model in studies of anti-inflammatory activity [39].

In this work, it was noticed that J774 macrophages were less sensitive to the cytotoxic effects of Pluronic F68 and DMSO and to high concentrations of ginger and rosemary extracts than the peritoneal macrophages. J774 is a murine macrophage cell line established from a spontaneous tumor developed in a female BALB/c mouse and some reports showed that the J774 cell line is a suitable model for the study of cytotoxicity and anti-inflammatory activity of different compounds to macrophages [39–41]. Tumor-originated cell lines are frequently resistant to a number of distinct external stimuli, what probably explains why the effects observed on the J774 cells were not as strong as the ones observed on the peritoneal macrophages.

Cytokines, including interleukins and tumor necrosis factor-α (TNF-α), as well as reactive oxygen and nitrogen species (ROS and RNS) have been the most studied inflammatory mediators. These substances initiate the inflammatory response, recruit and activate other cells to the site of injury and subsequently resolve the inflammatory process. ROS include hydrogen peroxide (H_2_O_2_), superoxide anion (O_2_^●−^), hydroxyl radical (OH^●^), single oxygen and lipid peroxides, while RNS include nitric oxide (NO) and species derived from NO, such as peroxynitrite (ONOO^−^). ROS and RNS at low levels contribute to cell signaling, mitochondrial respiration and biogenesis. However, when not properly controlled, high ROS and RNS levels cause DNA, lipid, and protein damage [42], and consequently, cell death may occur [43].

Inflammatory cells produce large amounts of nitric oxide (NO) when properly stimulated. NO is synthesized from L-arginine by a family of enzymes known as the nitric oxide synthases (NOS). In the immune system, the most important isoform is inducible NOS (iNOS). A wide variety of immune stimulants such as certain cytokines (IFN-γ, IL-1 and TNF-α) and/or bacterial endotoxins are potent inducers of iNOS [44]. Lipopolysaccharide (LPS) is an endotoxin and the major component of the outer membrane of Gram-negative bacteria, known for its various biological activities and strong stimulation of pro-inflammatory responses in macrophages [45, 46].

Nitric oxide released by iNOS from murine macrophages is cytostatic and cytotoxic for protozoan parasites, fungi and bacteria and despite the induction and activation of NOS along with excessive production of NO are common features of almost all infection-related diseases, acute or chronic inflammation may also result in the same outcomes [47]. Given that these mediators and cytokines play important roles in the pathogenesis of a vast number of human diseases [48], selective inhibitors of iNOS may in the future be useful in the treatment of several illnesses. In this regard, plant-derived substances as those evaluated in this work may be potential candidates.

In general, cytokines related with inflammatory response are not constitutively produced or are produced in low levels. However, the presence of appropriate stimuli, such as LPS, induces the production of pro-inflammatory cytokines such as interleukin-1β (IL-1β), interleukin-6 (IL-6), and TNF-α, leading to the initiation of an inflammatory response [49]. Based on these facts, the development of new biological therapies for inflammatory diseases has generally focused on the blockage of members of the inflammatory cascade, such as cytokines [50–52].

In this study, we have shown that both ginger and rosemary extracts dissolved in DMSO were able to inhibit LPS-induced NO production and were also capable to inhibit the production of TNF-α in murine elicited peritoneal macrophages. TNF-α and NO production were influenced in the same way by plant extracts and this could be explained by the fact that synthesis of NO is closely regulated by stimuli such as cytokine TNF-α. Endotoxins, such as LPS, and inflammatory cytokines, such as TNF-α and IL-1β, have been implicated in the expression of inducible nitric oxide synthase (iNOS), which produces NO in large amounts [53].

Some previous reports have documented the anti-inflammatory effects of ginger extracts [7, 54–56], but the results vary with cell type and inflammatory stimuli. Hong & Oh [54] and Shimoda et al [7] verified that ginger extracts or their constituents affect nitric oxide production by LPS-activated cells in a dose-dependent way for RAW264 cells. On the other hand, according to Jiang et al. [55], the bioactivity of ginger extracts may not be easily predicted. These authors verified that inhibition of LPS-induced prostaglandin E production in human histiocytes in vitro is possible with crude CH_2_Cl_2_ organic extracts of ginger, however, despite their components may act at several sites, the extracts were not nearly as effective at inhibiting TNF-α. In contrast, Surh [56] points that gingerol from the rhizome of ginger can suppress TNF-α production in mice and has potential for the therapy of TNF-related diseases. Lee and collaborators showed that ginger extracts from supercritical fluid extraction demonstrated free radical scavenging ability, reducing power and chelating property in dose-dependent manners [57].

Similarly, the biological activity of rosemary extracts and their main constituents has been extensively investigated, giving sometimes contradictory results. A methanol extract of rosemary and its hexane fraction reduced nitric oxide generation and blocked TNF-α production in LPS-stimulated RAW 264.7 cells. The inhibitory effects of methanol extract of rosemary re-extracted with others solvents (chloroform, ethyl acetate, n-butanol and water) on LPS-induced NO production was also analyzed. The effects of these extracts were not as effective as those of the methanol extract or of its hexane fraction [58]. Rosemary extract dissolved in DMSO showed to have a pronounced inhibitory effect on NO production by lipopolysaccharide (LPS)-activated RAW 264.7 macrophages [59]. On the other hand, no induction of inhibitory effects on the LPS-induced nitrite production by the same cell line was noticed when compared to the reference drug indomethacin [28]. Conversely, carnosic acid (CA), the main constituent of rosemary extract, inhibited LPS-induced oxidative/nitrosative stress in vivo by decreasing lipid peroxidation, protein carbonylation, and serum levels of nitric oxide [14]. Since NO has been suggested to play an important role in the physiology of inflammatory diseases, it is possible to infer that some vehicles used to deliver in vitro active compounds may therefore contribute negatively in this treatment.

Corroborating this result, Checker et al. [60] described the mechanism of anti-inflammatory activity of ursolic acid in activated T cells, B cells and macrophages. This acid is a pentacyclic triterpenoid carboxylic acid with antioxidant and anti-tumor properties and a constituent of rosemary extracts. Treatment of cells with ursolic acid significantly reduced the serum levels of pro-inflammatory cytokines [60].

Kuo et al. [61] also reported relevant results to support the potential use of rosemary extract obtained by supercritical carbon dioxide extraction and of its purified fractions as a nutraceutical formulation with inflammatory activity, showing marked suppression of LPS-induced production of NO and TNF-α by RAW 264.7 cells in a dose-dependent manner, particularly regarding carnosic acid. In addition, Tripathi et al. [62] reported a decrease of almost 10 fold in TNF-α production by macrophages obtained from C57BL/6 mice in the presence of ginger alcoholic extract plus LPS stimulation in comparison to macrophages stimulated with LPS alone. The authors showed that the production of LPS-induced IL-1β was completely inhibited in the presence of ginger alcoholic extract. However, these authors did not evaluate the effects of solvent alone nor of TNF-α / LPS stimulation in tumor cell lines [62]. Cattaneo and colleagues [63], reported the major components of the rosemary extract: rosmarinic acid, luteolin, apigenin, carnosol, caffeic acid and scutellarin. Hydroalcoholic extract of *Rosmarinus officinalis* reduced the proliferation of the human melanoma A375 cell line by a pro-oxidant activity of the extract. The antiproliferative activity was a property of the whole extract and seems to be resulting from multi-factorial effects of its components [63].

Our data showed that only the rosemary extract was able to significantly reduce the production of IL-1 in peritoneal macrophages cultured in the presence of LPS/IFN-γ. However, the ginger extract showed higher anti-inflammatory activity on the J774 tumor cell line than the rosemary extract. Thus, differences on NO, TNF-α and IL-1 production and cell proliferation between the macrophage models tested herein were observed. Most likely, these differences may be attributed to the origin of the cells. Tumor cells, such as the J774 cell line, may naturally present more resistance to external stimulus.

At a cellular level, the onset of pro-inflammatory reactions tends to start by release of early-responding cytokines such as IL-1α and -β and TNF-α. IL-1α/β and TNF-α subsequently regulate the expression of a variety of secondary cytokines and chemokines, including IL-6 and IL-8 [3]. Agents able to suppress TNF-α and IL-1 activity have potential for therapy of these TNF-α and IL-1-associated diseases. Monoclonal antibodies against these cytokines have emerged as an efficient treatment with many clinical benefits in experimental models of some diseases [64, 65]. However, the costs of antibody-based therapy are usually very high, thus supporting, justifying and stimulating the search for alternative approaches.

Neoplastic cells often over-express pro-inflammatory mediators including proteases, eicosanoids, chemokines and cytokines. Cytokines are major mediators of communication between cells in the inflammatory tumor microenvironment. Several cytokines such as macrophage migratory inhibitory factor (MIF), TNF-α, IL-6, IL-17, IL-12, IL-23, IL-10, and TGF-β have been associated with both experimental and human cancers and can either promote or inhibit tumor development. Then, inflammatory conditions precede development of malignancy in some cancers. In others tumors, oncogenic change drives a tumor-promoting inflammatory milieu [66].

Also, our results suggest that the lipid vesicles, DMSO and Pluronic, can protect the cells, probably due to changes in the form of absorption, distribution and cellular metabolism of hydrophobic molecules present in the culture medium. These results also can indicate that these vehicles may be rapidly absorbed and prevent the development the free radicals, acting as a radical scavenger that protects cells against compounds that increase intracellular levels of radical oxygen species. However, further assays are necessary to verify this hypothesis.

## Conclusions

Amongst the tested delivery vehicles, DMSO presented the lower cytotoxicity, followed by Pluronic F-68 and liposomes, provably due to differences in their form of absorption, distribution and cellular metabolism. Ginger and rosemary extracts obtained by supercritical CO_2_ extraction with yields of 2.4 and 2.9 % in mass, respectively, and with high antioxidant activity, were capable of inhibiting the production of LPS + IFN-γ-induced pro-inflammatory cytokines and NO production by peritoneal macrophages and J774 cells. Rosemary extracts had a more significant capability to inhibit IL-1 and TNF-α on macrophages, while ginger extracts showed the highest anti-inflammatory activity on the J774 tumor cell line. The anti-inflammatory effects of ginger and rosemary extracts were influenced by the delivery vehicles used and the type of cells employed.

The inhibition of cytokines and NO release by these extracts give support to their potential use as alternative approaches in the therapy of inflammatory diseases.

It is possible to conclude that the beneficial effects related with anti-inflammatory activity attributed to ginger and rosemary extracts could be associated with the inhibition of chemical mediators. New therapeutic opportunities based on their anti-inflammatory properties as well as their antioxidant and antineoplasic effects evaluated using the different delivery vehicles can be then be potentially envisaged.

However, since the extent of the anti-inflammatory effects may depend on the cell type and on the delivery vehicles used in the in vitro study, the achieved results should be interpreted with caution and must not be directly transposed to the likely response in vivo, since the bioavailability and metabolism of these bioactive compounds may be widely different in the body.
